# Artificial intelligence and patient narratives: A novel approach to assessing hope in patients with cancer

**DOI:** 10.3892/mi.2025.240

**Published:** 2025-05-07

**Authors:** Hakan Şat Bozcuk, Halil Göksel Güzel, Mustafa Özgür Arici, Mustafa Yildiz, Murat Koçer, Bilgeşah Kiliçtaş, Mehmet Artaç, Gökhan Karakaya, Hasan Şenol Coşkun

**Affiliations:** 1 Department of Medical Oncology, Medical Park Antalya Hospital, Antalya 07160, Turkey; 2 Department of Medical Oncology, SBU Antalya Training and Research Hospital, Antalya 07100, Turkey; 3 Department of Medical Oncology, Necmettin Erbakan University Hospital, Konya 04280, Turkey; 4 Department of Medical Oncology, Vakıf (ASV) Yaşam Hospital, Antalya 07300, Turkey

**Keywords:** cancer, artificial intelligence, neural network, hope, regression analysis

## Abstract

The present study aimed to evaluate the feasibility of patient-generated text and its interpretation by artificial intelligence (AI) as a valid correlate of hope levels in patients with cancer. For this purpose, four medical centers recruited consecutive patients with cancer and the patients were administered a questionnaire to collect data on patient characteristics and a shortened version of the Adult Trait Hope Scale (s-ATHS). Additionally, all participants provided written text on their hope levels, which was then analyzed by a deep neural network model. AI predicted hope labels, as well as numerous patient, disease and center features which were then associated with the scores from s-ATHS using univariate and multivariate gamma regression analyses. The present study comprised 461 patients with cancer, 194 (42.1%) of whom had metastatic disease. Multivariate gamma regression analysis identified three variables independently associated with hope index scores (s-ATHS): Treatment center (B=-0.09, Wald=4.77, P=0.029), Eastern Cooperative Oncology Group (ECOG) performance status (B=-0.09, Wald=47.41, P<0.001) and AI-predicted hope level (B=0.06, Wald=44.24, P<0.001). The results revealed that cases from one of the centers in the present study, a university hospital located in a different city than the other centers, exhibited higher hope levels. Additionally, a poorer ECOG performance status and lower AI-predicted hope levels were associated with reduced hope index scores (s-ATHS). On the whole, the present study demonstrates that AI-predicted hope levels are associated with hope index scores (s-ATHS), suggesting that monitoring AI-predicted hope levels may provide valuable insight in the practice of oncology.

## Introduction

Hope is a critical component of well-being and quality of life in various health contexts (1-3). In the realm of cancer, and in disease in general, hope is not only strongly associated with depression, but is also posited as a potential prognostic factor (4,5). Given its potential influence on key health outcomes, hope can be influenced by numerous factors related to cancer treatment and the disease itself (6,7). The available literature thus points at the multifaceted nature of hope, and underlines its multiple determinants, at the same time showing its clinical importance for various disease states, either to improve outcomes, improve quality of life or improve patient adaptation.

In the realm of cancer care, it is essential to evaluate both the functional capabilities and the psychological state of patients to deliver personalized treatments and provide comprehensive support. The Eastern Cooperative Oncology Group (ECOG) Performance Status is a widely recognized and standardized tool used to assess the functional ability of a patient and their capacity to perform everyday activities during cancer therapy (8). This assessment enables clinicians to determine appropriate treatment plans and predict patient outcomes based on functional limitations. Complementarily, the adult trait hope scale (ATHS) provides a concise yet effective measure of the inherent hopefulness of an individual by evaluating their motivational drive and perceived ability to devise strategies to achieve desired goals (9). The present study, by integrating these two dimensions of well-being, functional and psychological, aimed to construct a more comprehensive image of patient wellness and also explore the interplay between functional status and hope in the care of patients with cancer.

Artificial intelligence (AI) has revolutionized the practice of medicine, providing decision support systems and prognostic tools (10). However, its application to measuring hope levels in cancer care remains largely unexplored. While established hope indexes exist, the utilization of patient narratives and the data they generate have not been rigorously examined in this direction (11,12). Text data are a valuable means of expressing thoughts and emotions, and AI technology can unearth hidden patterns within these data that questionnaires may not potentially capture (13,14) (https://aws.amazon.com/what-is/text-analysis). However, in oncology in general, text data are not readily available or usable in predictive models. Thus, in the present study, the authors trained an AI model using patient-generated text data to explore the current significance of hope for cancer patients within their specific health contexts. Additionally, the present study evaluated whether the AI could predict hope levels alongside various patient and disease factors, including the ECOG performance status and scores from the shortened ATHS (s-ATHS).

## Patients and methods

### Patients, questionnaire administration and data collection

Over a 6-week period from August 1, 2023, to September 15, 2023, the present study enrolled consecutive patients from outpatient clinics in four medical oncology departments at various treatment centers (Departments of Medical Oncology at Medical Park Antalya Hospital, Antalya, Turkey; SBU Antalya Training and Research Hospital, Antalya, Turkey; Necmettin Erbakan University Hospital (KNEU), Konya, and Vakıf (ASV) Yaşam Hospital, Antalya, Turkey). Patients attending the outpatient clinics during this time period were approached by the medical staff, and were requested to take part in the study and complete the questionnaires. Data on patients accepting to participate in the study were collected and analyzed. Ethical committee approval from a teaching hospital was obtained in July, 2023 (Acceptance no. 2023-162, Antalya Training and Research Hospital, Antalya, Turkey); the approval was valid for all centers taking place in the study and the patients verbally consented to the study.

The participating medical centers included two private and two teaching hospitals (a university hospital and a research and training hospital), from two different cities (Medical Park Antalya Hospital, SBU Antalya Training and Research Hospital and Vakıf (ASV) Yaşam Hospital are in Antalya, Turkey, whereas Necmettin Erbakan University Hospital is in Konya, Turkey. All patients provided verbal informed consent and completed a two-page questionnaire. This questionnaire included questions related to the hope index, patient demographics, disease characteristics and treatment-related factors, along with an open-ended question as follows: ‘What does the word ‘hope’ currently mean to you in your life? Please describe your general hopes related to treatment and life, or the absence of such hopes, in a few sentences below’. The written responses of the patients to this question were evaluated by the AI model, as detailed in the ‘Artificial intelligence analysis of text data’ section below. In addition, a 4-item shortened version of the ATHS (s-ATHS) was also embedded into the questionnaire, which was shown to be as reliable as the original 12-item version (15). Patients independently completed the questionnaires, with occasional assistance from the medical staff. At the end of each day, questionnaire results were entered into standard databases and results from different centers were combined at the conclusion of the study. In order to ensure that the ethical implications of using AI in sensitive emotional assessments are responsibly addressed, data privacy and confidentiality were maintained through secure data storage protocols and anonymization of personal identifiers prior to analysis.

### Artificial intelligence analysis of text data

**Figure 1:**
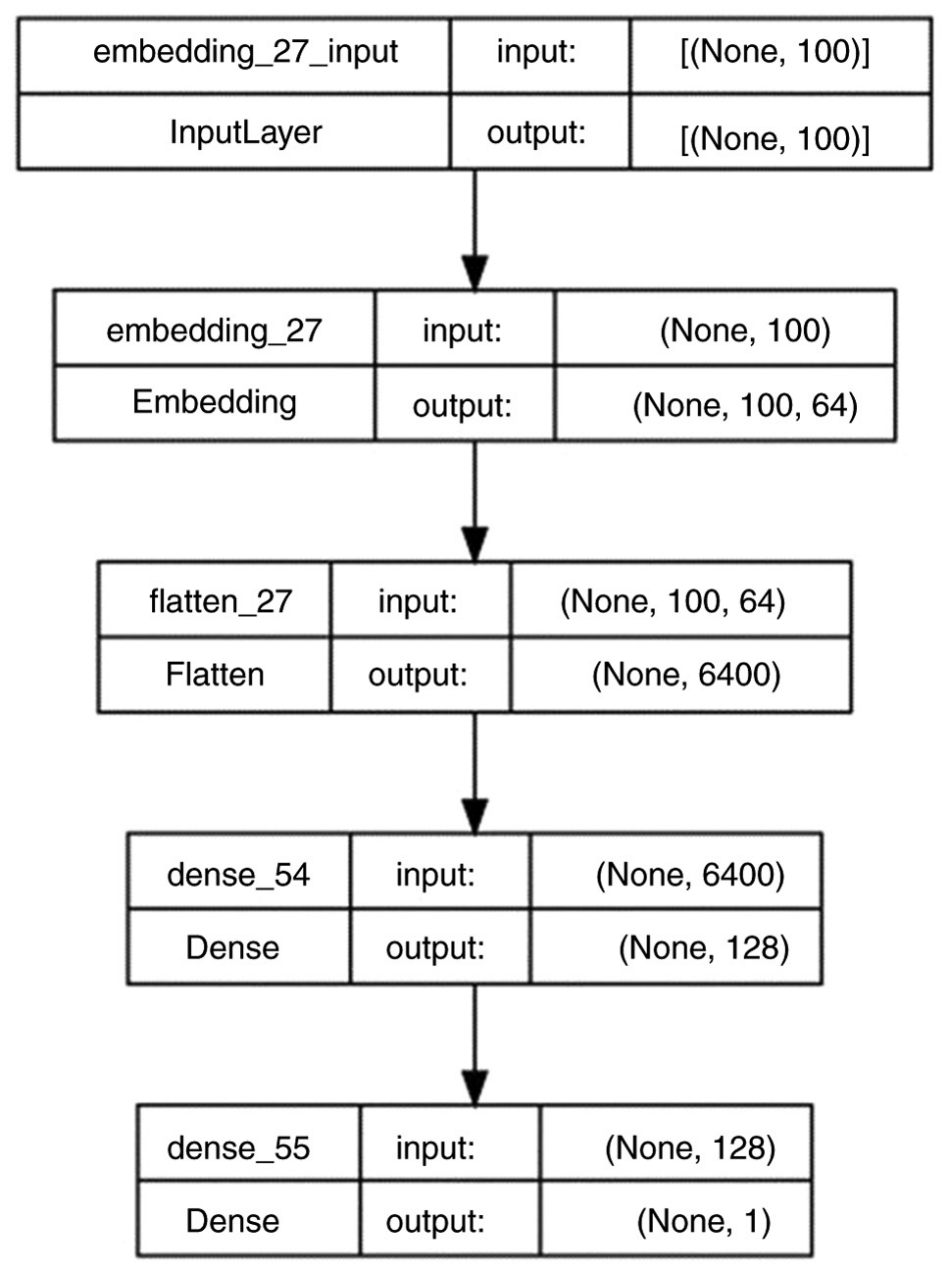
Deep neural network structure for the textual data. Characteristics and layers of the deep neural network for modelling textual data are shown.

The authors developed a deep neural network and trained it on patient-generated text data and manually labeled hope scores on a 10-point scale. In order to establish this, one author (HŞB) assigned hope scores, as blinded to the s-ATHS scores, to all patient text responses, which were then cross-validated by the other authors. The resultant neural network model was saved and then used to forecast individual AI-predicted hope levels for each participant. The AI code was written in Python and utilized libraries such as TensorFlow, Pandas, NumPy and NLTK (https://www.tensorflow.org; https://pandas.pydata.org; https://numpy.org, https://www.nltk.org). Text preprocessing included importing Turkish stop words from the NLTK library, converting the text to lowercase, and removing special characters and digits using regular expressions. Model accuracy was evaluated using the mean absolute error (MAE) and root mean squared error (RMSE) (16). The deep neural network consisted of five sequential layers from the Keras library and was trained for 80 epochs with a batch size of 256 (https://keras.io). Data were split into 80% for training and 20% for validation. The deep neural network structure is depicted in Fig. 1.

### Statistical analysis

Hope index scores (s-ATHS), calculated using standard methodology, were predicted through both univariate and multivariate gamma regression analysis. s-ATHS was subjected to logarithmic transformation due to the highly skewed nature of the response variable. Gamma regression was chosen for the regression analysis due to the fact that response variable was continuous, strictly positive, and skewed, even after log transformation. Independent variables included various patient, disease, and treatment factors, along with AI-predicted hope levels. Separate generalized linear models were constructed for each analysis. Variables with a P-value <0.10 in the univariate analyses were then included in the multivariate analyses; P-values <0.05 were considered to indicate statistically significant differences. SPSS 21.0 was employed for gamma regression analysis (https://www.ibm.com/spss).

## Results

### General characteristics

Out of the 475 recruited patients with cancer, 461 with complete data were used for AI model training and subsequent statistical analyses. The majority of the patients were female (57.7%), with a median age of 59 years. Metastatic disease was present in 42.1% of the patients. Breast, lung and colorectal cancer patients comprised 37.6% of cases, and the remaining majority of cases had varied solid cancers apart from breast, lung and colorectal cancers. The median s-ATHS score was 26 on a scale of 32, while AI-predicted hope levels had a median of 7.3 on a 10-point scale, with a RMSE of 1.5. The detailed patient characteristics are presented in Table I.

### AI model

**Figure 2:**
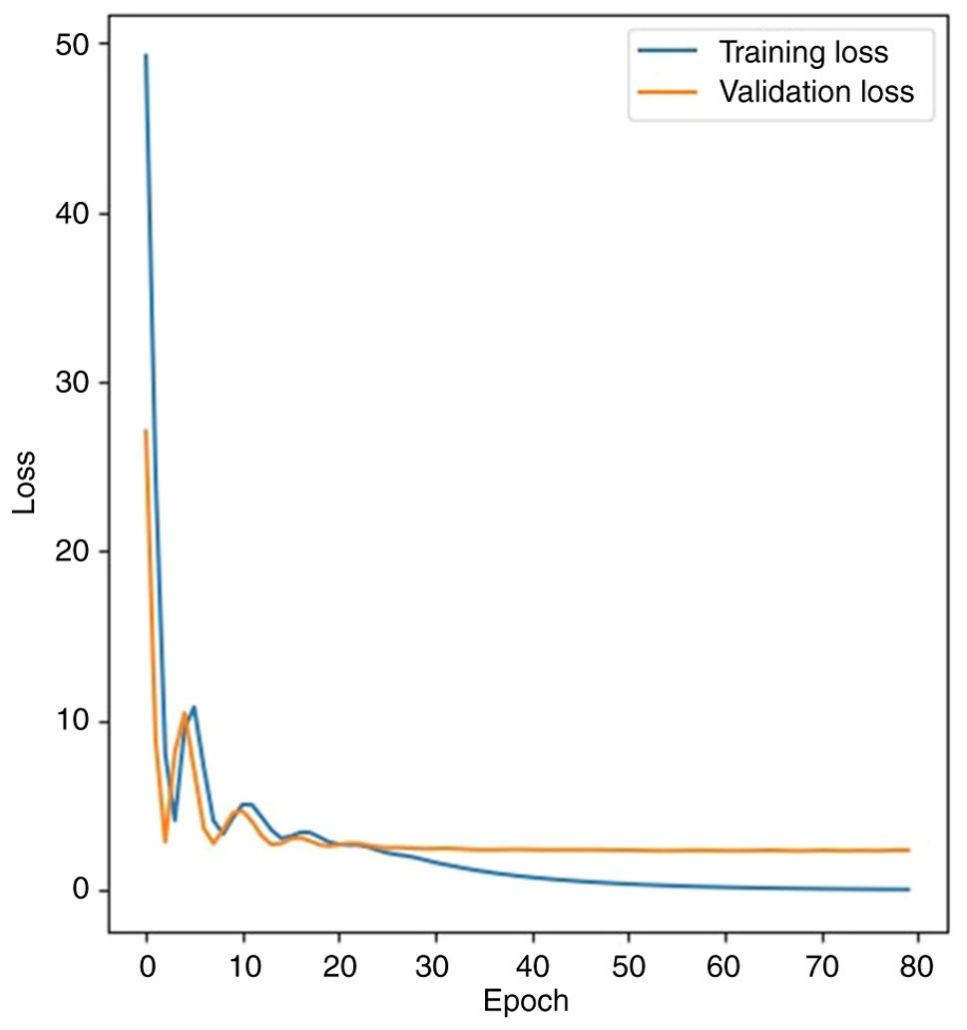
Loss function in the training and validation sets. Minimizing loss of function reflects the accuracy of the deep neural network.

The deep neural network effectively learned from patient text data, with the loss function [mean squared error (MSE)] decreasing from 52.29 to 0.08 in the training set, and from 36.09 to 2.34 in the validation set. The MAE figure dropped from 7.00 to 1.12 in the training set and from 5.80 to 1.18 in the validation set. The loss if function (MSE) changes in the training and validation sets are presented in Fig. 2.

### Determinants of hope

**Figure 3:**
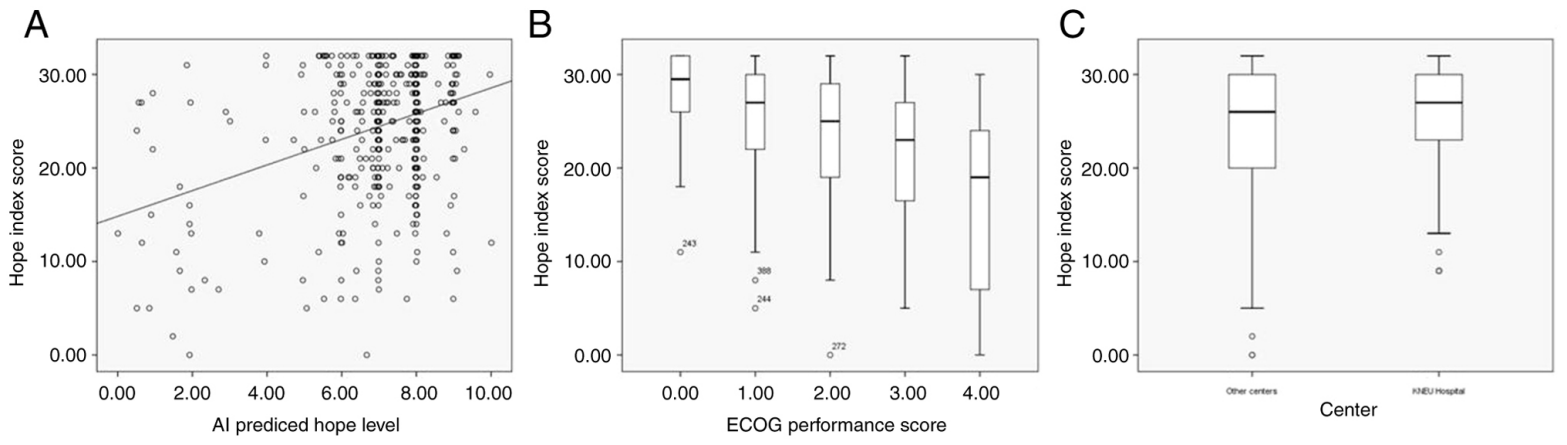
Key associations with the s-ATHS scores. (A) s-ATHS and AI predicted hope level, (B) s-ATHS and ECOG performance score, (C) s-ATHS and center. s-ATHS, shortened version of the adult trait hope scale; AI, artificial intelligence; ECOG, Eastern Cooperative Oncology Group.

Univariate gamma regression analysis revealed several factors associated with s-ATHS scores, including the treatment center, age, cancer type, cancer stage, ECOG performance status, education, income and AI-predicted hope level with varying P-values (<0.10). In a multivariate gamma regression model, three factors remained independently significant: The treatment center (c3 vs. others, Wald=4.77, P=0.029), ECOG performance status (Wald=47.41, P<0.001) and AI-predicted hope level (Wald=44.24, P<0.001). The associations of these three factors with s-ATHS are presented in Table II and Fig. 3. Of note, although stage was statistically significant in the univariate gamma regression model (Wald=5.22, P=0.022), it lost significance in the multivariate testing stage (Wald=0.05, P=0.818). The median s-ATHS scores in stage 4 cases was 25, and in earlier stages (stage 1 to 3) it was 27.

As regards to the treatment center variable, c3 was the only center from a university hospital and was located in a different city compared to the other three centers. The other three centers were from private and research and training hospitals, located in another city. The center c3 (KNEU) was different in a number of ways compared to other centers with a younger patient age, a greater number of female patients, a greater number of patients diagnosed with breast and colorectal centers, a greater utilization of active treatment, and a lower level of university education. The selected patient features with respect to the different centers are presented in Table II. Patients from c3 had higher s-ATHS values. Additionally, an increasing ECOG score was associated with lower s-ATHS values; median s-ATHS figures were 29.5 in ECOG 0, 27 in ECOG 1, 25 in ECOG 2, 23 in ECOG 3 and 19 in ECOG 4 classes. Lastly, the AI-predicted hope level was positively associated with s-ATHS. Further details of the univariate and multivariate analyses are presented in Table III.

## Discussion

The present study introduces a novel AI method for assessing hope in patients with cancer using patient-generated narratives. This approach may provide a practical means of evaluating hope and potentially other dimensions of well-being with implications for research and clinical practice. The present study incorporated a diverse patient population from various high-volume cancer treatment centers, encompassing different cancer diagnoses and disease stages. The heterogeneity of patient enrollment in the present study suggests that the findings may be generalized to various cancer patient populations and different treatment and disease settings. Notably, three common cancer types, breast, lung and colorectal cancers, comprised 37.6% of the included cases, reflecting the regional distribution of cancer incidence. Additionally, the substantial proportion of metastatic patients, at 42.1% of the study population, underscores the applicability of the resulting AI model to different groups of cancer patients with respect to type of diagnosis and extent of disease.

Various hope indexes and their versions have been long-standing tools for measuring hope levels, particularly in the context of chronic diseases and cancer (9,10,17,18). In the present study cohort, the median hope index score of 26 on a 32-point scale highlight an above average hope level in the study respondents. To train the AI model, manually labeled hope labels were utilized rather than s-ATHS scores, as training the model on the same entity it is intended to predict would introduce bias. This approach proved successful, as AI-predicted hope labels trained on patient-generated text data closely mirrored and predicted s-ATHS.

In addition to hope and performance status, other psychosocial variables, such as depression, anxiety and social support may also play critical roles in shaping patient well-being and treatment outcomes. Future studies of AI models in the field of psycho-oncology could incorporate textual markers of these psychosocial factors, enriching the understanding of the multidimensional patient experience. Previous research has demonstrated that emotional distress and lack of social support can negatively affect both hope and survival in patients with cancer (19,20). By including these variables, AI models can potentially serve as a more comprehensive predictor of patient well-being and even long-term survival.

Several associations identified in the present study warrant further investigation. For instance, it would be noteworthy to examine whether why one center (c3) exhibited higher hope scores (s-ATHS) compared to the others. While the exact reasons for this remain unclear, factors specific to the center, such as being a university hospital, or local characteristics, such as city-level influences, or unnamed factors, may play a role. Although some differences related to this center were detected compared to the other centers in terms of a younger patient age, a greater predominance of the female sex, more frequent diagnoses of breast and colorectal cancer, a heavier utilization of active treatment and a lower level of education, the authors consider that none of these factors alone are sufficient to explain higher hope scores at this center, as these factors have separately been evaluated in the regression analyses. Additionally, it was found that, while advanced-stage disease was negatively associated with hope in the univariate regression analysis, this association did not remain significant in the multivariate model. This finding is in accordance with the findings in the study by Cohen *et al* (21), where it was shown that hope can be maintained even in the latest stages of advanced-stage cancer. This highlights the complex interplay of multiple factors influencing hope among patients with cancer with diverse diagnoses and disease stages.

Of note, in the present study, the use of AI to assess hope through patient-generated narratives builds upon recent advancements in AI-based psychosocial research. Studies, such as that by de Hond *et al* (22) demonstrated the effectiveness of deep learning models in predicting depression from textual data and electronic records in oncology settings, highlighting the potential of AI to capture complex emotional states. By validating AI-predicted hope labels against the established s-ATHS, the present study corroborates these advancements and also extends them by specifically focusing on hope as a critical psychosocial factor. This comparison underscores the promising role of AI in enhancing psychosocial assessments in clinical practice.

AI is ushering in a revolution in medicine, facilitating the development of predictive and prognostic models, decision support systems, and sense-making from complex and heterogeneous data sources (23). While the present study marks the initial use of AI for comprehending textual data for hope in cancer care, this approach holds promise for application in other disease settings and for assessing various dimensions of patient well-being, whether psychological or physical. Such AI models can serve exploratory, explanatory, predictive, or prognostic purposes. The authors are particularly interested in exploring the potential prognostic utility of this approach and intend to follow-up with other patients to investigate whether AI-predicted hope levels have predictive value. Currently, the limited literature suggests that diminished hope levels may predict poorer prognoses in various health contexts, such as advanced-stage cancer and coronary heart disease (5,24,25). In the case that the AI-predicted hope level demonstrated herein is able to provide prognostic information, it will be noteworthy to determine whether it surpasses s-ATHS scores in its association with prognoses.

The present study has several limitations which should be mentioned. Although the inclusion of 461 participants provided a solid foundation for demonstrating the feasibility of the AI-based approach, larger datasets are required to capture more nuanced and complex associations among variables. Expanding the study to include data from thousands of patients could significantly enhance the explanatory and predictive power of the model. Additionally, future research is required to examine specific patient subgroups to obtain deeper insight into the associations within these categories. For instance, analyzing non-metastatic and metastatic groups within a single cancer type could yield valuable information.

Another limitation is the lack of the assessment of depression, which may act as a confounding factor influencing hope levels. Depression is known to reduce the expression of hope and overall psychological well-being, potentially biasing the results, particularly among patients with more advanced stages of cancer (26). Consequently, some of the observed associations between hope and other variables may be partially attributable to underlying depressive symptoms. Future studies are thus warranted to incorporate measures of depression to more accurately isolate the effect of hope and enhance the validity of psychosocial assessments in cancer care.

In conclusion, the present study used a novel AI approach to assess hope in patients with cancer by leveraging patient narratives. This approach provides a practical and insightful method for assessing hope and other aspects of well-being, with implications for research and clinical practice. Looking ahead, AI-driven models could not only evaluate hope, but could also help guide personalized psychosocial interventions for patients. For example, personalized treatment plans could be developed based on AI assessments of narrative-driven emotions, helping clinicians identify patients who may benefit from pharmacological intervention, psychological support, counseling, or spiritual care. Additionally, as AI technology advances, it may become feasible to integrate real-time data from patient narratives shared via mobile health apps or social media platforms, allowing for continuous monitoring of hope and other psychosocial variables throughout the cancer journey.

In summary, assessing hope using textual data with the help of AI technology has the potential to help us understand better cancer patients and how they cope with their disease. Using this approach, it is considered that the association of hope with the wellbeing of patients with cancer and prognostic outcomes can be evaluated.

## Figures and Tables

**Table I:** Patient demographics.

Characteristic	No. of patients	%	Median	Mean	Minimum	Maximum
Total no. of patients	461	100				
Center						
Antalya Training and Research Hospital	220	47.7				
Antalya Medical Park Hospital	49	10.6				
Konya Necmettin Erbakan University	124	26.9				
Antalya Vakıf Yaşam Hospital	68	14.8				
Sex						
Female	266	57.7				
Male	195	42.3				
Age, years			59	57.7	21	87
ECOG performance status			1	1.5	0	4
Education						
Primary or secondary school	261	56.6				
Lycee	88	19.1				
University	112	24.3				
Income						
Poor	120	26				
Average	325	70.5				
High	15	3.3				
Marital status						
Married	360	78.1				
Not married	101	21.9				
Type of cancer						
Breast cancer	81	17.6				
Lung cancer	45	9.8				
Colorectal cancer	47	10.2				
Other types of cancer	288	62.5				
Disease stage						
1 to 3	267	57.9				
4	194	42.1				
Time since diagnosis						
<6 months	146	31.7				
6 to 12 months	101	21.9				
>1 year	214	46.4				
Treatment status						
Active treatment	363	78.7				
No active treatment	98	21.3				
Expression of feelings			3	2.8	1	4
s-ATHS score			26	24.5	0	32
Hope label^a^			8	7.2	0	10
AI-predicted hope level			7.3	7.1	0	10

Patient characteristics, as well as hope related variables are defined.

^a^Manually labeled hope score. s-ATHS, shortened version of the adult trait hope scale; ECOG, Eastern Cooperative Oncology Group.

**Table II:** Selected features of patient from the different centers.

Patient features	KNEU center (c3)	Other centers
Age (median)	55.31	58.61
Female sex (%)	66%	55%
Diagnosis of breast or colorectal cancer (%)	49%	25%
Utilization of active treatment (%)	96%	72%
Graduated from university (%)	13%	29%
Hope index scores (mean)	25.88	24.04

**Table III:** Predictors of hope in patients with cancer.

		Univariate analysis				Multivariate analysis			
Parameter		B	Wald	df	P-value	B	Wald	df	P-value
Center^a^									
	c1 [ATRH (center 1) vs. others]	0.08	6.04	1	0.014	0.04	1.12	1	0.29
	c2 [AMPH (center 2) vs. others]	-0.08	2.69	1	0.101				
	c3 [KNEU (center 3) vs. others]	-0.07	3.87	1	0.049	-0.09	4.77	1	**0.029**
Age, years		-0.003	5.22	1	0.022	-0.002	1.99	1	0.158
Sex		0.02	0.25	1	0.618				
Type of cancer^b^									
	ct0 (breast cancer vs. others)	-0.02	0.14	1	0.708				
	ct1 (lung cancer vs. others)	0.02	0.15	1	0.697				
	ct2 (colorectal cancer vs. others)	-0.12	5.27	1	0.022	-0.04	0.68	1	0.41
Cancer stage		0.07	5.22	1	0.022	-0.01	0.05	1	0.818
Active treatment		-0.05	2.09	1	0.148				
Expression of feelings		-0.008	0.18	1	0.675				
Time since diagnosis		0.01	0.51	1	0.475				
ECOG performance status		-0.1	56.32	1	<0.001	-0.09	47.41	1	**<0.001**
Education		0.04	4.43	1	0.035	0.01	0.39	1	0.533
Income		0.08	5.75	1	0.016	0.01	0.23	1	0.631
Marital status		-0.003	0.01	1	0.934				
AI-predicted hope level		0.06	44.76	1	<0.001	0.06	44.24	1	**<0.001**

Univariate and multivariate analyses of the association of various features on hope level (s-ATHS) in patients with cancer.

^a^c1, c2, c3 are dummy variables for center, and

^b^ct0, ct1, ct2 are dummy variables relating to the type of cancer. Values in bold font indicate a statistically significant difference (P<0.05). s-ATHS, shortened version of the adult trait hope scale; ECOG, Eastern Cooperative Oncology Group.

## Data Availability

The data generated in the present study may be requested from the corresponding author.
